# Characteristics of *Stenotrophomonas maltophilia* infection in children in Sichuan, China, from 2010 to 2017

**DOI:** 10.1097/MD.0000000000019250

**Published:** 2020-02-21

**Authors:** Lili Wang, Wei Zhou, Yang Cao, Chunsong Yang, Hanmin Liu, Ting Chen, Lina Chen

**Affiliations:** a Division of Pediatric Pulmonology and Immunology, West China Second University Hospital, Sichuan University; b Key Laboratory of Birth Defects and Related Diseases of Women and Children (Sichuan University), Ministry of Education; c Clinical Laboratory, West China Second University Hospital, Sichuan University; d Department of Pharmacy, West China Second University Hospital, Sichuan University, Chengdu, Sichuan, China.

**Keywords:** children, clinical characteristic, drug susceptibility, *Stenotrophomonas maltophilia*

## Abstract

*Stenotrophomonas maltophilia* (*S. maltophilia*) is an important nosocomial bacterial pathogen. However, the clinical features of children with *S. maltophilia* infection, the predisposing factors, and the antibiotic susceptibility of the bacteria have not been fully evaluated.

In this study, the data of children with *S. maltophilia* infection from the West China Second University Hospital of Sichuan University (Chengdu, China) between July 2010 and October 2017 were collected and analyzed. The clinical features of enrolled children, the predisposing factors, and the antibiotic susceptibility were reported.

In total, infection of *S. maltophilia* was identified in 128 patients. Most of these patients were under 1 year old (67.2%) and were mainly diagnosed as pneumonia (69%). A large proportion had underlying diseases (45.3%), received immunosuppressive therapy (53.1%), had undergone invasive operations (41.4%), had a history of carbapenem antibiotics use within 7 days before culture acquisition (54.7%), history of intensive care unit (ICU) hospitalization within previous 30 days (34.4%), and other risk factors. In particular, invasive operation (95% confidence interval [CI]: 1.125–14.324, *P* = .032), especially mechanical ventilation (95% CI: 1.277–20.469, *P* = .021), and ICU admission (95% CI: 1.743–22.956, *P* = .005) were independent risk factors for the children to develop severe *S. maltophilia* infection. As for antibiotic susceptibility, trimethoprim sulfamethoxazole (TMP-SMX), piperacillin tazobactam, ticarcillin clavulanate, and ceftazidime exhibited strong antibacterial activities against *S. maltophilia,* the susceptibility rates were 97.5%, 86.7%, 92.9%, and 81.5%, respectively.

We report the clinical features of children with *S. maltophilia* infection, the predisposing factors and the antibiotic susceptibility. TMP-SMX can continue to be the first choice for the treatment of *S. maltophilia* infection. Piperacillin tazobactam, ticarcillin clavulanate, and the third generation cephalosporins can be used as alternative drugs.

## 1 Introduction

*Stenotrophomonas maltophilia* (*S. maltophilia*) is a Gram-negative, nonfermentative organism.^[1]^ It is one of the opportunistic pathogens of nosocomial infections, causing such serious infections in immunocompromised patients as pneumonia, septicemia, as well as infections of the skin and soft tissue, surgical wounds, and the urinary tract.^[1,2]^ Pediatric mortality due to *S. maltophilia* bacteremia was reported to be 6% to 40%.^[3,4]^ Due to aminoglycoside acetyl-transferase and enzymes that inactivate erythromycin and genes encoding efflux pumps, *S. maltophilia* strains are intrinsically resistant to a variety of antibiotics.^[5]^ Therefore, selection of an appropriate antimicrobial regimen for the treatment of *S. maltophilia* infection is a challenge for clinicians.

Thus far, most clinical studies of *S. maltophilia* have focused on the adult population, and only a limited number of studies describing the infection in children have been reported.^[6–8]^ Overall, there is a dearth of data on the clinical characteristics of this infection in Chinese children. In this study, we aimed to close this gap and explore the clinical features of children with *S. maltophilia* infection, the predisposing factors, and the antibiotic susceptibility. We reasoned that the results should be helpful for early recognition and initiation of appropriate treatment of *S. maltophilia* infection in the clinical setting.

## 2 Materials and methods

### 2.1 Subjects and ethics statement

**Figure 1:**
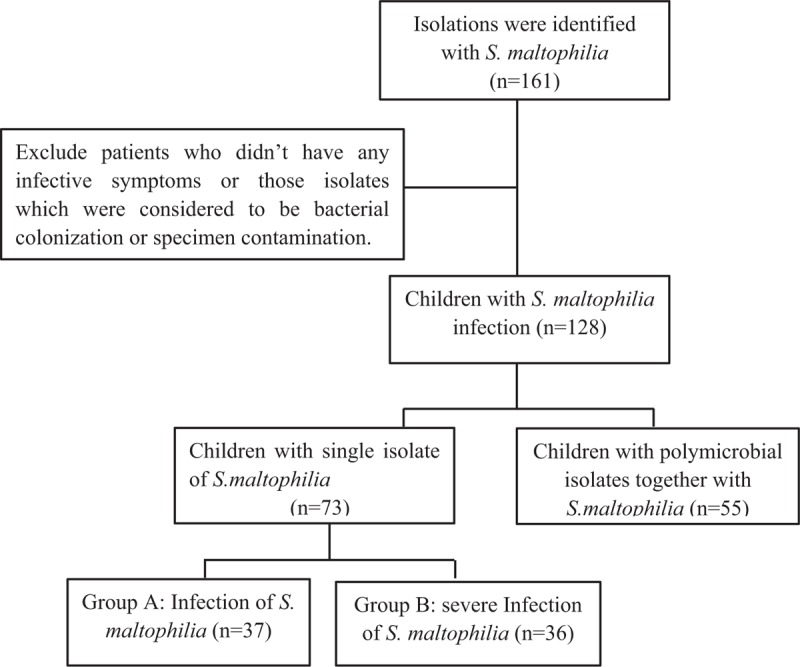
Study inclusion and exclusion criteria applied for patients.

This study was conducted retrospectively at the West China Second University Hospital, Sichuan University (Chengdu, China). Data were collected between July 2010 and October 2017 from electronic medical records. The cultures that contained viable *S. maltophilia* were identified. Patients who satisfied the following criteria of *S. maltophilia* infection were included for further analysis (Fig. 1).

The Institutional Review Board/Ethics Committee affiliated with West China Second University Hospital, Sichuan University, approved this study, which was performed in accordance with the ethical standards of the Declaration of Helsinki.

### 2.2 Definitions

#### 2.2.1 Infection of *S. maltophilia*

The criteria for diagnosis of *S. maltophilia* infection were as follows:

(1) Site of isolation: isolates from nonrespiratory sites were included for analysis. The respiratory isolates were included only if they were from bronchoalveolar lavage fluid (BALF), tracheal intubation secretion or high quality sputum, that is, the number of leukocytes in the sputum smear was more than 25 /high power field (HPF), and the epithelial cell number was less than 10/HPF, or the ratio of leukocyte to epithelial cell was no less than 10.^[9]^ A BALF culture was considered positive when the number of *S. maltophilia* colonies was ≥10^4 CFU/mL. As for the high quality specimen of sputum or tracheal secretion, it was also considered significant if *S. maltophilia* isolate was the sole bacteria or the dominant bacteria in the mixed flora.
(2) Manifestations: patients should have clinical symptoms or signs of the corresponding site infection (such as shortness of breath, dyspnea, headache, etc), with associated laboratory results such as increased leukocytes and increased percentage of neutrophils or increased procalcitonin level, and elevated nucleated cells in the cerebrospinal fluid.^[10]^ Conversely, if patients did not have any infective symptoms or associated laboratory manifestations, their isolates were considered to be bacterial colonization or specimen contamination, and therefore, not included in the analysis. Appropriate empirical therapy was defined as microorganism susceptibility to one of several antimicrobial agents administered within 72 hours after the onset of bacterial infection.

#### 2.2.2 Severe infection of *S. maltophilia*

The severity of illness was assessed by the Acute Physiology and Chronic Health Evaluation II score. The Charlson comorbidity index was used as an aggregate measure of comorbidities.^[7,11]^ Those who met any of the following criteria were classified as suffering severe *S. maltophilia* infection:

(1) Patients died during this hospitalization; the attributable mortality (bacteremia-related death) was judged by 2 infectious diseases physicians, when the patient had no other identifiable reason for death.^[6]^ If the death occurred within 7 days of isolation of *S. maltophilia*, it was considered related to the infection regardless of the presence of comorbid conditions that could potentially account for death.^[12]^
(2) Patients were diagnosed as severe pneumonia, respiratory failure, heart failure or multiple organ dysfunction, and the association with *S. maltophilia* was evaluated by 2 infectious diseases physicians.

#### 2.2.3 Underlying diseases

Renal diseases included nephrotic syndrome, renal insufficiency, and so on. Respiratory diseases included bronchopulmonary dysplasia, tracheobronchomalacia, and tracheobronchial stenosis. Heart diseases mainly included congenital heart diseases. Autoimmune diseases included systemic lupus erythematosus, and so on. Neurologic diseases included epilepsy, neonatal hypoxic and ischemic encephalopathy, and so on. Gastrointestinal diseases mainly included gastroesophageal reflux.

### 2.3 Microbiology

An automatic identification system, the Vitek2 system (bioMe’reux, Marcy l’Etoile, France) was used to identify isolates of *S. maltophilia*. The antimicrobial susceptibilities were measured with ATB PSE 5, 25 STRIPS (bioMe’reux, Marcy l’Etoile), and were interpreted according to the latest Clinical Laboratory Standard Institute M100 guideline.

### 2.4 Data collection and statistical analysis

The age, sex, manifestations, auxiliary examination, diagnosis, treatment, and other clinical information of the children were collected. Data were analyzed using the SPSS 19.0 software package (IBM, Armonk, NY). Continuous variables were compared using Student *t* test or the nonparametric Mann–Whitney *U* test, and categorical variables were compared using the Chi-squared (*χ*^2^) or Fisher exact test. A logistic regression analysis was performed to study the associations between variables and disease severity. Two-sided *P* values of <.05 were considered statistically significant.

## 3 Results

### 3.1 Isolation rates and clinical characteristics of patients

A total of 161 children had positive isolates of *S. maltophilia* between July 2010 and October 2017. Among them, infection of *S. maltophilia* was identified in 128 patients (128/161, 79.5%), whereas 10 (10/161, 6.2%) isolates were considered as colonization or contamination and 23 (23/161, 14.3%) were of undetermined origin. The respiratory tract was identified as the main infection site. The proportion of infecting and colonizing or contaminating isolates varied according to the site of isolation (Table 1).

**Table 1:**
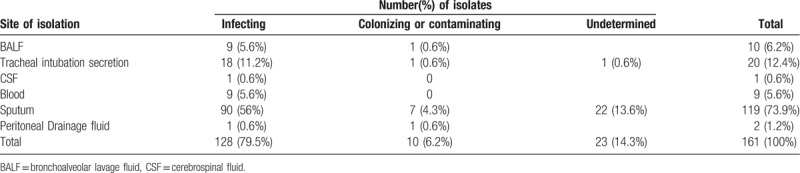
Site of isolation of *Stenotrophomonas maltophilia* (n = 161).

During the study period, patients with *S. maltophilia* infection were mainly newborns and infants younger than 1-year-old, and 57% (73/128) of them were male. The mean length of hospitalization was 20 days (range, 1–134 days). The clinical manifestations of children infected with *S. maltophilia* were diverse. Besides fever and other systemic symptoms, respiratory symptoms such as dyspnea and cough were the most common manifestations. Overall, 69% of the children were primarily diagnosed as pneumonia, and 45.3% had underlying diseases (mainly due to premature birth, heart diseases, and hematological malignancy). In addition, 53.1% of the children received immunosuppressant therapy, among them 45.3% were given glucocorticoids. The ratio of patients who underwent invasive operations was 41.4%. The most common procedures included mechanical ventilation (37.5%), and central venous catheterization (9.4%). More than one-third of children had a history of intensive care unit (ICU) admission within the previous 30 days, and 54.7% of the patients received carbapenem within 7 days before culture acquisition (Table 2).

**Table 2:**
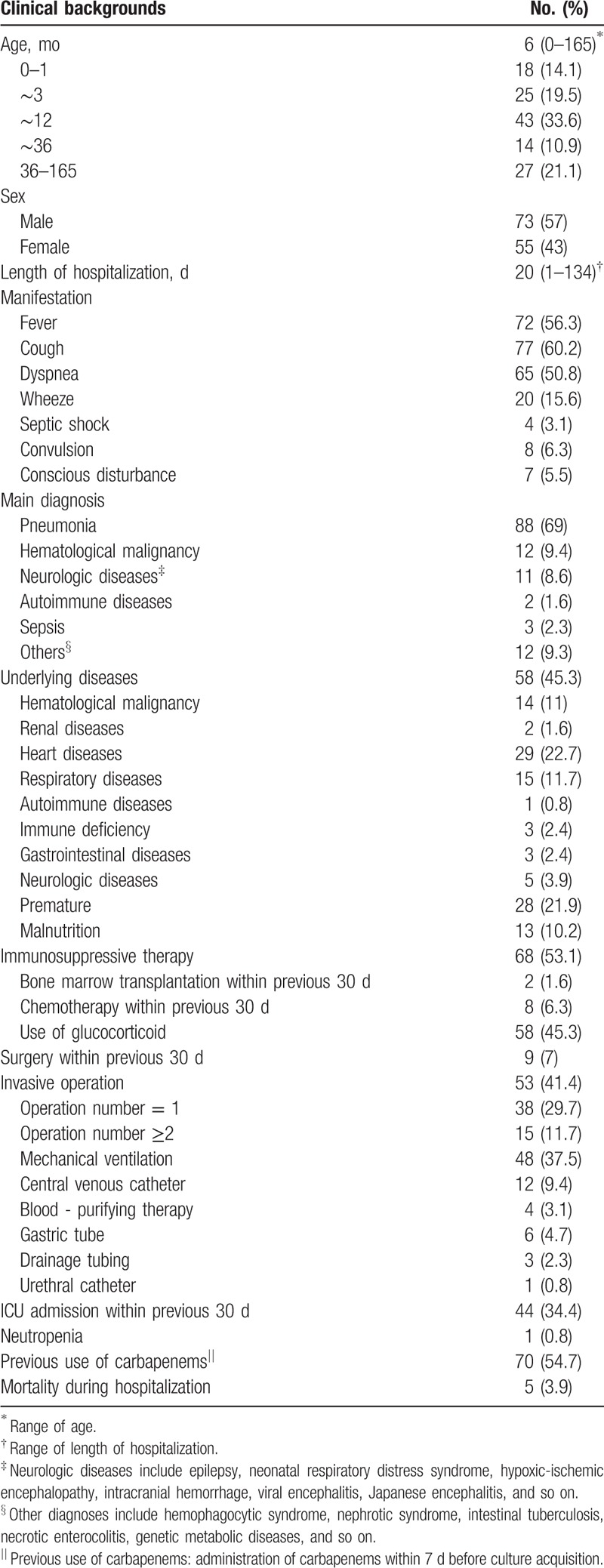
Demographic and clinical characteristics of patients with *Stenotrophomonas maltophilia* infection (n = 128).

The antibiotic susceptibility of *S. maltophilia* isolates was summarized in Table 3. The highest antibiotic sensitivity were shown towards trimethoprim sulfamethoxazole and ticarcillin clavulanate. Piperacillin tazobactam and ceftazidime also had substantial antibacterial activities against the bacterium, the susceptibility rates being 86.7% and 81.5%, respectively. All isolates were resistant to imipenem and meropenem, whereas 75% of the isolates were resistant to ampicillin sulbactam.

**Table 3:**
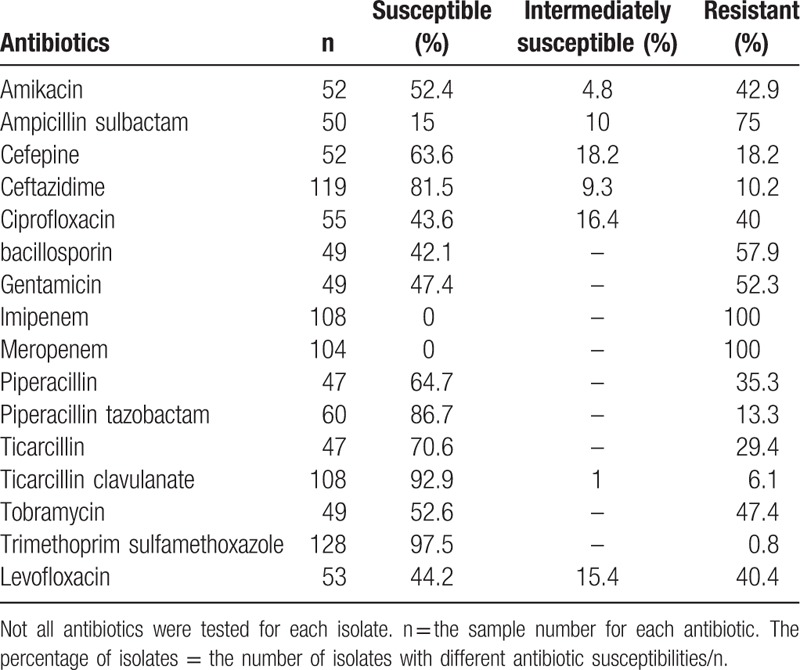
Antibiotic susceptibility of *Stenotrophomonas maltophilia* isolates.

### 3.2 Risk factors associated with severe *S. maltophilia* infection

Co-isolated bacterium, along with *S. maltophilia*, were found in 25 specimens (19.5%); of these, *Klebsiella pneumoniae* was the most common one (5.5%), followed by *Acinetobacter baumannii* (4.7%) and *Pseudomonas aeruginosa* (3.9%). Once the patients infected with multiple pathogens (such as other bacterium, fungus, mycoplasma, etc) were excluded, there were 73 children left with monomicrobial isolation. Based on the severity of the disease, these 73 patients were divided into severe and nonsevere groups, and the characteristics of the 2 groups were compared. There were no significant differences in age, sex, length of hospitalization, underlying comorbidities, and immunosuppressive therapy between the 2 groups. However, there were significantly higher rates of invasive operations (24.3% vs 52.8%, *P* = .012), mainly mechanical ventilation (10.8% vs 44.4%, *P* < .001), ICU admission (13.5% vs 44.4%, *P* = .004), and previous use of carbapenems (37.8% vs 69.4%, *P* = .007) in the severe group compared with the nonsevere group (Table 4).

**Table 4:**
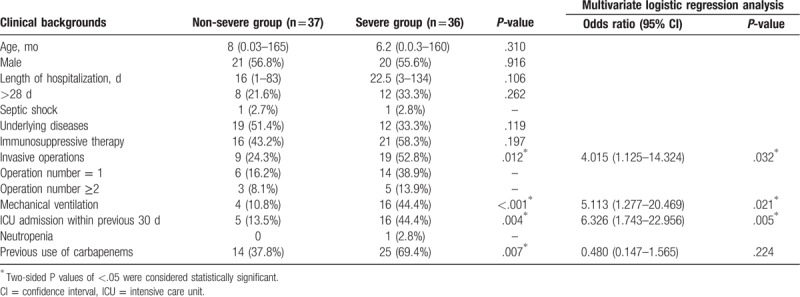
Factors associated with severe disease in patients with single isolate of *Stenotrophomonas maltophilia* (n = 73).

Through logistic regression analysis, we determined factors that were significantly associated with severe *S. maltophilia* infection. The results of the multivariate analysis indicated that invasive operations (95% CI: 1.125–14.324, *P* = .032), especially mechanical ventilation (95% CI: 1.277–20.469, *P* = .021), and ICU admission (95% CI: 1.743–22.956, *P* = .005) were independent risk factors for the development of severe *S. maltophilia* infection (Table 4).

## 4 Discussion

*S. maltophilia* is emerging as an opportunistic pathogen among hospitalized pediatric patients. To understand the characteristics of *S. maltophilia* infection in children, we conducted this retrospective analysis and analyzed the antibiotic susceptibility as well as risk factors for severe diseases.

*S. maltophilia* infection mainly causes respiratory symptoms such as dyspnea, cough, and so on. Patients with *S. maltophilia* infection tended to have prolonged hospitalization, underlying diseases, immunosuppressive therapy, invasive operation, history of ICU admission, and previous use of carbapenems, which were in agreement with the results of studies in adults.^[13,14]^ The most common underlying illness identified in our study were heart diseases, and the most common invasive operation was mechanical ventilation. *S. maltophilia* can adhere to medical materials (eg, tracheal intubation) with its biofilm and increases the chances of lower respiratory tract infection, which may be the reason why patients with *S. maltophilia* infection tend to have a history of medical invasive operation.^[22]^

In our study, the majority of children with *S. maltophilia* infection suffered from pneumonia (n = 117). And 59% (69/117) of these children developed severe pneumonia. The reasonable treatment of *S. maltophilia* infection is of great important.

Treatment of *S. maltophilia* infection is difficult, in part because the bacteria is resistant to a variety of antimicrobial agents. Trimethoprim-sulfamethoxazole (TMP-SMX) has been generally effective, based on in vitro susceptibility assays and reports of clinical outcomes.^[15–17]^ However, an increasing number of studies have reported the resistance of *S. maltophilia* to TMP-SMX,^[7,18]^ which presents a major challenge to clinical physicians. Therefore, we considered it important to determine the resistance rate of *S. maltophilia* to commonly used antibiotics, especially TMP-SMX in children. Fortunately, we found that *S. maltophilia* was highly susceptible to TMP-SMX, which was consistent with most studies published thus far.^[8,19,20]^ This suggests that TMP-SMX could still be the first choice for the treatment of *S. maltophilia* infection. Furthermore, a large proportion of isolates were also susceptible to ticarcillin clavulanate. A variety of studies have reported high resistance rates of *S. maltophilia* to cephalosporin antibiotics.^[7,20,21]^ However, ceftazidime showed substantial antibacterial activity against the bacteria in our study. The reason for the difference is currently unclear. The irrational use of cephalosporins maybe 1 reason. In addition, the sensitivity rate to piperacillin tazobactam was also found to be high. We, therefore, suggest that the third generation cephalosporins, as well as piperacillin tazobactam and ticarcillin clavulanate can be used as alternative drugs to patients who cannot tolerate TMP-SMX. Of note, *S. maltophilia* exhibits high-level intrinsic resistance to carbapenem antibiotics because of the production of the versatile L1 type β-lactamase (also called “carbapenemase”), which is capable of hydrolyzing carbapenem antibiotics.^[19]^

To our knowledge, few studies have analyzed the risk factors for children to develop severe *S. maltophilia* infection. In the present study, we have analyzed the clinical characteristics of children with severe infection, and identified the associated risk factors. To exclude the effect of polymicrobial infection on the results of the study, we only analyzed the children with monomicrobial *S. maltophilia* infection. Invasive operations (especially mechanical ventilation), use of carbapenems within 7 days before culture acquisition, and ICU admission within the previous 30 days were found to be associated with severe infection. Multivariate logistic regression analysis identified invasive operations (mainly consisting mechanical ventilation) and ICU admission as independent risk factors for the development of severe infection. This may be due to the fact that the clinical application of ventilator weakens the cough reflex and the mucosal cilia clearance function, and in parallel, promotes proliferation of the bronchial glands and increased secretion, which in turn increases the chances of respiratory infection.^[22]^ Thus, for children with long term ICU hospitalization and invasive operations such as mechanical ventilation, the clinicians should keep a particularly wary eye on *S. maltophilia* infection. Rational use of broad-spectrum antibiotics such as the carbopenems and practicing meticulous hand hygiene are also advised.

There were some potential limitations of our study. First, it was a single center study with a moderate sample size, which may not be generalizable. Second, because of the retrospective design, selection and observational bias may have affected the results. A more elaborate, match controlled study may be conducted in the future to further confirm our results.

In conclusion, TMP-SMX can continue to be the first choice for the treatment of *S. maltophilia* infection, while piperacillin tazobactam, ticarcillin clavulanate, and the third generation cephalosporins can be used as alternative drugs. To prevent the severe diseases, clinicians should use antibiotics more rationally, ensure good management of sterilization practices, and isolate children with high-risk factors.

## Author contributions

**Conceptualization:** Lili Wang, Lina Chen.

**Data curation:** Lili Wang, Wei Zhou, Yang Cao, Ting Chen.

**Formal analysis:** Lili Wang, Chunsong Yang.

**Funding acquisition:** Hanmin Liu, Lina Chen.

**Investigation:** Lili Wang.

**Methodology:** Lili Wang, Wei Zhou.

**Software:** Lili Wang.

**Supervision:** Hanmin Liu, Lina Chen.

**Visualization:** Lili Wang.

**Writing – original draft:** Lili Wang.

**Writing – review and editing:** Lili Wang, Lina Chen.
